# Comprehensive Evaluation of Bisphenol A Toxicity Reveals Neurobehavioral, Metabolic, and Reproductive Impairments in *Girardia tigrina*

**DOI:** 10.1002/tox.70039

**Published:** 2026-01-19

**Authors:** Raquel de Jesus Selestrino, Felipe Schaly, Welligton Luciano Braguini

**Affiliations:** ^1^ Departamento de Biologia, Laboratory of Experimental Biochemistry and Toxicology (BioTox) Universidade Estadual do Centro Oeste (Unicentro) Guarapuava Brazil; ^2^ Cell Biology Department Federal University of Paraná (UFPR) Curitiba Brazil

**Keywords:** acetylcholinesterase, bisphenol A, *Girardia tigrina*, LC_50_, locomotor activity, oxidative stress, reproduction

## Abstract

Bisphenol A (BPA), a common industrial chemical used in plastics and consumer products, is increasingly detected in aquatic environments, raising serious concerns about its potential ecological impacts. This study evaluated the sublethal and acute toxic effects of BPA on the freshwater planarian *Girardia tigrina* , a recognized model for neurotoxicity and regenerative biology. Acute exposure revealed a time‐dependent decrease in LC_50_ values, from 53.18 μM at 24 h to 22.38 μM at 96 h. Behavioral assays showed significant locomotor impairment, with activity reduced by 36.7% and 57.2% at 1.0 and 5.0 μM, respectively. The LC_50_ for movement inhibition was estimated at 2.79 μM after only 5 min of exposure. Stereotyped movements intensified with concentration and duration. Reproduction was markedly affected, with fecundity and fertility reduced by 96.7% and 100% at 2.5 μM; no hatchlings emerged at 1.0 or 2.5 μM. Glycogen reserves dropped by 42.9% at 2.5 μM, indicating metabolic disruption. Marked oxidative stress responses were observed, with superoxide dismutase and catalase activities increasing by over 220%. Glutathione S‐transferase activity was elevated across all concentrations. Acetylcholinesterase activity showed a biphasic response—strong inhibition at lower doses (−67.8% at 0.1 μM), followed by stimulation at 1.0 μM (+56.9%)—suggesting cholinergic disruption. Collectively, these results demonstrate that BPA disrupts multiple physiological systems in planarians at environmentally relevant levels, underscoring the need for more comprehensive environmental monitoring and the adoption of safer chemical alternatives to protect aquatic biodiversity.

## 1 Introduction

Bisphenol A (BPA, 2,2‐bis(4‐hydroxyphenyl)propane; CAS 80‐05‐7) is a synthetic compound extensively used in the production of polycarbonate plastics and epoxy resins, commonly found in food packaging, thermal paper, and other consumer goods [1, 2, 3]. Despite its industrial utility, BPA poses significant environmental and health concerns due to its ability to leach into food, water, and ecosystems, especially under conditions of heat or acidity [4, 5, 6, 7].

In recent years, BPA has faced increasing global regulatory scrutiny due to its well‐documented endocrine‐disrupting properties. In 2023, the European Food Safety Authority (EFSA) drastically reduced the tolerable daily intake (TDI) of BPA to 0.2 ng/kg body weight/day based on immunotoxicology evidence [8]. This revision effectively bans BPA in food contact materials under current EU legislation. Previously, the specific migration limit (SML) set by Commission Regulation (EU) No 10/2011 was 50 μg/kg. In aquatic environments, allowable BPA levels vary across countries: Canada and China have established limits of 10 μg/L in drinking water and industrial effluents, respectively [9, 10, 11], while the EU's revised Drinking Water Directive proposes a 2.5 μg/L threshold for endocrine‐disrupting substances in potable water sources [12].

Despite such regulatory advances, BPA continues to be frequently detected in surface waters, sediments, and aquatic organisms due to its historical use, environmental persistence, and ongoing release from recycled materials [13, 14, 15]. Recent biomonitoring studies have reported BPA in human urine, serum, and even placental tissues, including in populations residing in areas with partial bans, highlighting its continued relevance as a contaminant of concern [16, 17]. Environmentally relevant concentrations of BPA, ranging from nanograms to low micrograms per liter, have been shown to elicit sublethal effects in non‐target aquatic species, raising concerns about potential long‐term ecological impacts.

Moreover, BPA remains a reference compound for evaluating the toxicity of its structural analogs, such as bisphenol S (BPS) and bisphenol F (BPF), which are increasingly used as BPA substitutes but remain insufficiently characterized from an ecotoxicological perspective [15]. Continued investigation into the biological effects of BPA, especially at environmentally relevant concentrations, is thus essential not only to refine regulatory thresholds but also to generate comparative data for the ecological risk assessment of emerging bisphenols [18].

The freshwater planarian *Girardia tigrina* has emerged as a powerful model organism in ecotoxicology due to its ecological relevance, high sensitivity to pollutants, and unique biological features. As a benthic invertebrate widely distributed across the Americas, *G. tigrina* is in direct contact with sediment‐bound contaminants and plays a critical role in freshwater ecosystems [19, 20]. It possesses a centralized nervous system with approximately 10 000 neurons and shares many neurotransmitters with vertebrates, enabling reliable assessments of neurotoxicity, behavior, and regeneration [21, 22, 23]. The species exhibits remarkable regenerative capacity and reproductive plasticity, allowing for multi‐endpoint studies in a single model. Furthermore, planarians do not require ethical committee approval in most jurisdictions, making them suitable for high‐throughput toxicity screening [24, 25].

Several studies have demonstrated that planarians respond sensitively to sublethal concentrations of environmental pollutants by exhibiting altered behaviors, impaired regeneration, metabolic disturbances, and reproductive deficits [21, 26, 27, 28]. Behavioral endpoints such as locomotion and stereotyped seizure‐like movements (pSLMs) are reliable markers of neurotoxicity, while glycogen content serves as a proxy for metabolic disruption. Reproductive output (fecundity and fertility) and enzymatic biomarkers—such as superoxide dismutase (SOD), catalase (CAT), glutathione S‐transferase (GST), and acetylcholinesterase (AChE)—offer insights into oxidative stress and cholinergic pathway perturbations.

This study presents a comprehensive ecotoxicological evaluation of BPA's sublethal effects on *G. tigrina* , integrating behavioral, metabolic, biochemical, and reproductive endpoints. Median lethal concentrations (LC_50_) were determined across multiple exposure durations, and sublethal responses were assessed at environmentally realistic concentrations. By employing an alternative and sensitive invertebrate model, this work contributes to a more mechanistic understanding of BPA toxicity in aquatic systems and supports the development of refined risk assessment frameworks that encompass subtle, yet ecologically significant effects of endocrine‐disrupting chemicals (EDCs).

## 2 Materials and Methods

### 2.1 Chemicals

BPA (≥ 99% purity, CAS: 80‐05‐7), acetylthiocholine iodide (ASCh; ≥ 98%, CAS: 1866‐15‐5), 5,5′‐dithiobis‐(2‐nitrobenzoic acid) (DTNB; ≥ 98%, CAS: 69‐78‐3), d‐(+)‐glucose (≥ 99.5%, CAS: 50‐99‐7), silver(I) sulfate (Ag_2_SO_4_; ≥ 99%, CAS: 10294‐26‐5), bovine serum albumin (BSA; fraction V, ≥ 98%, CAS: 9048‐46‐8), nitroblue tetrazolium chloride (NBT; ≥ 98%, CAS: 298‐83‐9), reduced l‐glutathione (GSH; ≥ 98%, CAS: 70‐18‐8), 1‐chloro‐2,4‐dinitrobenzene (CDNB; ≥ 97%, CAS: 97‐00‐7), hydrogen peroxide (H_2_O_2_; 30% w/w aqueous solution, CAS: 7722‐84‐1), hydroxylamine hydrochloride (NH_2_OH·HCl; ≥ 99%, CAS: 5470‐11‐1), Tris(hydroxymethyl)aminomethane (Tris; ≥ 99.9%, CAS: 77–86‐1), ethylenediaminetetraacetic acid disodium salt dihydrate (EDTA; ≥ 99%, CAS: 6381‐92‐6), ethylene glycol‐bis(β‐aminoethyl ether)‐N,N,N′,N′‐tetraacetic acid (EGTA; ≥ 98%, CAS: 67‐42‐5), and Triton X‐100 (laboratory grade, CAS: 9002‐93‐1) were purchased from Sigma‐Aldrich (St. Louis, MO, USA). All other reagents used were of analytical grade and obtained from standard commercial sources.

### 2.2 Planarian Culture Conditions and Maintenance

The triclad flatworm *G. tigrina* (Girard) was used as the experimental model in this study. Specimens were maintained in BPA‐free polypropylene containers, a material widely employed in aquatic toxicology due to its chemical stability and low potential for leaching plastic additives. The absence of BPA in these containers was verified by manufacturer specifications and ensured that no background contamination would interfere with the experimental endpoints. Planarians were cultured in artificial pond water (APW) at 21°C ± 1°C under near‐darkness, at the Laboratory of Experimental Biochemistry and Toxicology (BioTox), located at Universidade Estadual do Centro Oeste (Unicentro), Guarapuava, Brazil. APW was prepared using distilled water with 6 mM NaCl, 0.13 mM CaCl_2_, and 0.12 mM NaHCO_3_ [29, 30]. Animals were fed twice a week with bovine liver sourced from a certified local butcher. The liver was rinsed in distilled water, manually portioned, and stored in sterile aliquots at −20°C without any chemical additives. Although the liver was not analytically tested for BPA contamination, all animals were subjected to a 7‐day fasting period prior to testing, and both culture media and containers were replaced 48 h after the last feeding. These procedures were implemented to reduce the likelihood of dietary or residual BPA affecting the experimental outcomes. All assays were carried out under controlled laboratory conditions and independently verified by at least two observers. After completion of all observations, planarians were humanely euthanized by immersion in 2% hydrochloric acid, a well‐established and rapid method for planarian euthanasia [19, 20], which induces immediate tissue dissolution and irreversible loss of motility.

### 2.3 Experimental Design and Concentration Selection

The concentrations of BPA used in this study (0.1, 0.5, 1.0, 2.5, and 5.0 μM) were selected based on two criteria: (i) reported environmental levels in surface waters and sediments, and (ii) the acute toxicity threshold (96 h LC_50_) previously determined for *G. tigrina* (22.38 μM, Table 1). Environmental monitoring studies have reported BPA concentrations ranging from low nanograms per liter to several micrograms per liter, particularly in urbanized or industrial regions [10, 32]. The selected concentrations in the present study ranged from approximately 4–224 times lower than the LC_50_, thereby representing sublethal exposure levels that are both environmentally relevant and suitable for detecting early biochemical and behavioral alterations.

#### 2.3.1 Exposure Conditions

All exposures were conducted under static conditions in BPA‐free polypropylene Petri dishes (90 mm diameter × 15 mm height), with each dish containing 20 mL of test solution. Five BPA concentrations were tested: 0.1, 0.5, 1.0, 2.5, and 5.0 μM, prepared by dilution from a stock solution in ultrapure water. A vehicle control was included. For each treatment and control group, three biological replicates were used, and each replicate consisted of five adult *G. tigrina* planarians (*n* = 15 per group). Animals were randomly distributed into dishes and exposed for 96 h at 20°C ± 1°C in complete darkness (0 lx) to avoid photodegradation of the compound and to maintain the animals in their preferred photophobic state, minimizing baseline stress for the subsequent biochemical (glycogen and enzymes) and reproductive analyses. Test solutions were renewed every 24 h to maintain chemical stability and water quality. All planarians were starved for 7 days prior to exposure and acclimated to experimental conditions for 24 h before the start of treatments.

Note on illumination for behavioral assays: While the 96‐h exposure was conducted in darkness, the subsequent locomotor (pSLM) test was performed under controlled illumination. The pSLM assay was conducted under uniform light (~400 lx) to ensure total environment visibility for tracking.

Although the bovine liver used for feeding was sourced from a certified local supplier and handled without additives, its BPA content was not analytically verified. This represents a practical limitation commonly encountered in invertebrate toxicology studies, where routine chemical screening of biological feed sources is often unfeasible. However, to minimize the likelihood of dietary BPA contamination influencing the experimental outcomes, a standardized seven‐day fasting period was implemented prior to all exposures. In addition, culture media and containers were replaced 48 h after the last feeding. These procedures, seven‐day fasting and the renewal of culture medium before testing, are widely applied in planarian toxicology [20, 21, 22, 33] and are broadly accepted as effective strategies to prevent residual BPA from confounding the interpretation of sublethal toxicity endpoints.

### 2.4 Determination of Median Lethal Concentration (LC_50_ )

A preliminary range‐finding test was conducted to identify the concentration range in which BPA caused partial mortality in *G. tigrina* over a 96‐h period. This initial screening employed widely spaced concentrations (1, 10, 25, 50, and 100 μM) to capture the general lethality threshold and guide subsequent refinement. Based on the observed mortality patterns, a definitive LC_50_ assay was then performed using eight more narrowly spaced concentrations (18, 20, 22, 24, 26, 28, 30, and 32 μM) to accurately estimate the 96 h LC_50_. Each concentration was tested in triplicate with five animals per replicate, under the same conditions described for the sublethal exposure experiment.

The LC_50_ value was calculated using probit analysis with 95% confidence intervals [31]. Each treatment was replicated six times to ensure statistical reliability. Planarians were monitored every 24 h for movement and mortality throughout the 96‐h exposure period. Individuals that exhibited no detectable movement were classified as dead and promptly removed from the test solutions. Mortality was defined as the absence of any spontaneous or mechanically induced movement after 60 s of observation, including gentle stimulation with a pipette tip. This criterion is widely used in planarian toxicity testing. Additionally, individuals classified as dead were kept in solution for a further 12 h to confirm irreversible loss of motility. In several cases, autolysis occurred subsequently, validating the lethality assessment. Median lethal concentrations (LC_50_) were calculated based on mortality observed at 24, 48, 72, and 96 h post‐exposure. A limitation of this study is that autolysis was not used as the primary mortality endpoint, although secondary confirmation frequently occurred.

Although BPA stock solutions were made in ultrapure water, the final exposure media were prepared by diluting these stocks directly into APW, ensuring that the ionic strength remained identical to that of the controls. As a result, planarians were never exposed to deionized water at any stage of the experiment. To confirm that both control and BPA treatments shared comparable osmotic characteristics, the osmolarity of all test solutions was assessed with a digital refractometer, which consistently yielded values of approximately 11–12 mOsm/kg. While refractometry provides an indirect estimate of osmolarity, it is widely accepted for low solute solutions and offers sufficient accuracy to ensure physiological comparability across treatments. In addition, the pH of all solutions, controls and BPA alike, was monitored throughout the exposure period and remained within a physiologically acceptable range (6.5–7.2). Maintaining this stability helped minimize pH‐dependent variations in BPA toxicity and ensured consistent exposure conditions for all groups.

### 2.5 Planarian Locomotor Velocity (pLmV) Assessment

The mobility assays were designed to evaluate the locomotor responses of *G. tigrina* to BPA following short‐term (24 h) exposure. Each BPA treatment was replicated in triplicate at three independent experiments to ensure robust data collection. Planarians were exposed to BPA (0.1, 0.5, 1.0, 2.5, and 5.0 μM) for 24 h based on the results from the acute toxicity tests. The mobility of the planarians was measured in polypropylene Petri dishes (BPA‐free; 60 mm diameter × 15 mm height) filled with 10 mL of the APW. For locomotion quantification, a sheet of graph paper (5 × 5 mm grid) was positioned beneath the Petri dish to provide a precise reference for tracking. The mobility assessment was conducted at a controlled temperature of approximately 21°C ± 1°C. The planarian was gently placed in the center of the plate, and the mobility was expressed as the number of lines crossed and re‐crossed by the planarian during 5 min of filming with a high‐speed NEOCoolcam‐Webcam camera with a varifocal zoom lens. To minimize potential optical distortion, the camera was mounted directly above and perpendicular to the arena, and the grid lines were confirmed to be parallel to the video frame edges during setup. The effective concentrations causing a 50% reduction in mobility (inhibitory concentration 50 [IC_50_]) were subsequently calculated at respective times (1–5 min), providing a fast and reliable indicator of acute toxicity in response to BPA exposure.

### 2.6 Quantification of Planarian Seizure‐Like Movements (pSLM)

Planarians (*n* = 20) were placed in 50 mL tubes with experimental solutions of BPA (0.1, 0.5, and 1.0 μM), and were closed for 5 min, 2 h, 4 h, and 24 h to induce pSLMs. After these times, planarians were transferred to a polypropylene Petri dish (BPA‐free; 60 mm diameter × 15 mm height), with APW in the absence of any other compounds and measured for pSLM during 10 min of filming with NeoCoolcam‐Webcam camera: “headbop” (bobbing head while moving), “corkscrew” (spiral movement while in motion), “tailtwist” (twisting of the tail), “headswing” (head movement while tail is anchored), and “squirming” (jerking or scrunching of the body) as described by Pagán et al. and Raffa and Desai [34, 35].

All behavioral scoring was conducted independently by two trained observers using high‐resolution video recordings obtained with a NeoCoolcam webcam and analyzed offline, which allowed careful frame‐by‐frame inspection whenever subtle movements required closer evaluation. Inter‐observer reliability was assessed in a randomized 25% subset of the video recordings, following standard methodological recommendations for behavioral and toxicological studies [36, 37]. Using a subsample within the 20%–30% range is widely recognized as sufficient to ensure scoring consistency without imposing unnecessary redundancy [38]. The resulting Cohen's kappa value (*κ* = 0.87) falls within the “almost perfect agreement” range according to Landis and Koch [39], confirming high reliability between observers. In cases where discrepancies emerged, the observers jointly reviewed the corresponding video segment to establish a consensus, ensuring uniform criteria across the dataset. Given that certain stereotypic behaviors may overlap, such as a tail twist occurring simultaneously with body squirming, each behavioral category was evaluated independently according to its defining movement characteristics, following established guidelines in behavioral analysis.

### 2.7 Planarian Reproduction Assessment

The reproduction was evaluated by monitoring populations of randomly selected individuals and the concentrations of BPA tested were 0.1, 0.5, 1.0, and 2.5 μM. The planarians were placed in 120 × 20 mm glass Petri dishes containing 100 mL of APW or APW with BPA at the indicated concentrations. For each concentration, eight planarians were used. Cocoons and hatchlings produced in each treatment were counted daily and transferred to isolated containers to determine the time to hatch. Fecundity was measured as the number of cocoons produced by sexually reproducing animals. Cocoons that did not hatch within 40 days were considered unviable. Fecundity (Fc) and fertility (Fr) indexes for each species were calculated as:

$$\mathrm{Fc}=C_{i}/N_{i}$$

where *C*_*i*_ is the number of cocoons produced by planarians in a given week and *N*_*i*_ is the number of individuals within a sampled population.

$$\mathrm{Fr}=H_{i}/N_{i}$$

where *H*_*i*_ is the number of hatchlings produced by individual cocoons in a given week, and *N*_*i*_ is the number of individuals within a sampled population in the experiment [40].

### 2.8 Glycogen Quantification in Planarians

The planarians were placed in 120 × 20 mm glass Petri dishes containing 100 mL of APW or APW supplemented with BPA at the indicated concentrations. They were exposed to BPA (0.1, 0.5, 1.0, 2.5, and 5 μM) for 7 days without feeding. Glycogen levels were assessed in planarians (*n* = 10; 120–160 mg) ground with 5 mL of deproteinizing solution [41] using a Potter–Elvehjem homogenizer. The homogenate was heated in a boiling‐water bath for 15 min, cooled, and centrifuged (3000 rpm, 5 min). The supernatant (1 mL) was mixed with 3 mL of 36 N H_2_SO_4_, heated for 5 min, and analyzed at 520 nm. Glycogen concentration was determined using a glucose standard curve. The deproteinizing solution contained 5 g trichloroacetic acid and 100 mg Ag_2_SO_4_ in 100 mL water, stored in an amber bottle at 4°C.

### 2.9 Enzyme Assays

Planarians were exposed to 0.1, 0.5, and 1.0 μM BPA for 24 h in 100 × 15 mm glass containers. After 24 h of exposure to BPA, the planarians were rinsed three times with APW. Enzymatic extracts were prepared by homogenizing planarians in a Potter–Elvehjem tissue homogenizer using 0.1 M phosphate buffer (pH 7.5) with 0.1% (v/v) Triton X‐100, except for SOD and AChE assays, where tissues were homogenized on ice in 60 volumes (v/w) of Tris‐citrate buffer (50 mM Tris, 2 mM EDTA, 2 mM EGTA, pH 7.4, adjusted with citric acid). The homogenates were centrifuged at 12 500 × *g* for 20 min at 4°C, and the supernatants were collected as the enzyme source for all assays. The activities of SOD, CAT, GST, and AChE were measured using a Shimadzu UV/Vis 340 spectrophotometer. Four independent experiments were performed for each enzyme and BPA concentration, with all assays conducted in triplicate.

#### 2.9.1 SOD Assay (E.C. 1.15.1.1)

SOD activity was measured by the inhibition of NBT photoreduction [42], and nitrite levels were determined using the diazo‐coupling method [43]. The reaction mixture (1.0 mL) contained 50 mM sodium carbonate (pH 10.2), 24 μM NBT, 0.1 mM EDTA, 0.03% (v/v) Triton X‐100, and 1 mM hydroxylamine. The absorbance at 560 nm was recorded every 30 s for 10 min under aerobic conditions. One unit of SOD activity was defined as the amount of enzyme required to inhibit 50% of NBT photoreduction, and the results were expressed as *U* × mg^−1^ of protein.

#### 2.9.2 CAT Assay (E.C. 1.11.1.6)

CAT activity was determined spectrophotometrically by measuring the decrease in absorbance at 240 nm, reflecting the decomposition of hydrogen peroxide [44, 45]. The reaction mixture consisted of 100 μL of enzyme extract and 2900 μL of 12 mM H_2_O_2_ in 50 mM potassium phosphate buffer (pH 7.0) containing 1 mM EDTA. Measurements were taken in quartz cuvettes (10 mm path length) every 15 s for 3 min. Enzyme activity was calculated using the molar extinction coefficient of H_2_O_2_ (*ε* = 40 M^−1^ cm^−1^) and expressed as *U* × mg^−1^ of protein.

#### 2.9.3 GST Assay (E.C. 2.5.1.18)

GST activity was determined following the method of Habig et al. [46], using 1‐chloro‐2,4‐dinitrobenzene (CDNB) as the substrate, prepared in 80% ethanol [46]. The reaction mixture contained 0.1 M potassium phosphate buffer (pH 6.9), 1 mM reduced glutathione (GSH), 1 mM CDNB, and 100 μL of enzyme extract. A control reaction without enzyme was used to account for non‐enzymatic activity. The conjugation of CDNB with GSH was monitored by the increase in absorbance at 340 nm for 5 min at 25°C. Enzyme activity was expressed as *U* × mg^−1^ of protein.

#### 2.9.4 AChE Assay (E.C. 3.1.1.7)

AChE activity was measured by the hydrolysis rate of 0.8 mM acetylthiocholine (ASCh) in a reaction mixture containing 50 mM Tris buffer (pH 7.0), 0.1% BSA, and 0.33 mM DTNB, as described by Ellman et al. [47]. The formation of the thiolate dianion from DTNB was monitored at 412 nm for 10 min, with readings every 30 s [47]. Controls without enzyme were included to assess non‐enzymatic hydrolysis. Enzyme activity was expressed as units of acetylthiocholine hydrolyzed per milligram of protein (*U* × mg^−1^ of protein).

### 2.10 Protein Determination

The protein content was quantified using the Bradford method with bovine serum albumin as a standard [48].

### 2.11 Statistical Analysis

All statistical analyses were conducted to evaluate the toxicity and behavioral effects of BPA on *G. tigrina* . The median lethal concentrations (LC_50_) at 24, 48, 72, and 96 h were calculated using probit regression analysis, an established method for estimating acute toxicity [31]. The IC_50_ for planarian locomotor activity, defined as the concentration at which velocity was reduced to 50% of the control (normalized to 100%) after 5 min of exposure, was estimated using nonlinear regression. Group comparisons were performed using two‐way analysis of variance (ANOVA), followed by Dunnett's post hoc test for multiple comparisons when significant differences were observed. All statistical analyses were conducted using GraphPad Prism (version 8.01 for Windows; GraphPad Software Inc., San Diego, CA, USA), and significance was defined at *p* < 0.05.

## 3 Results

**FIGURE 1:**
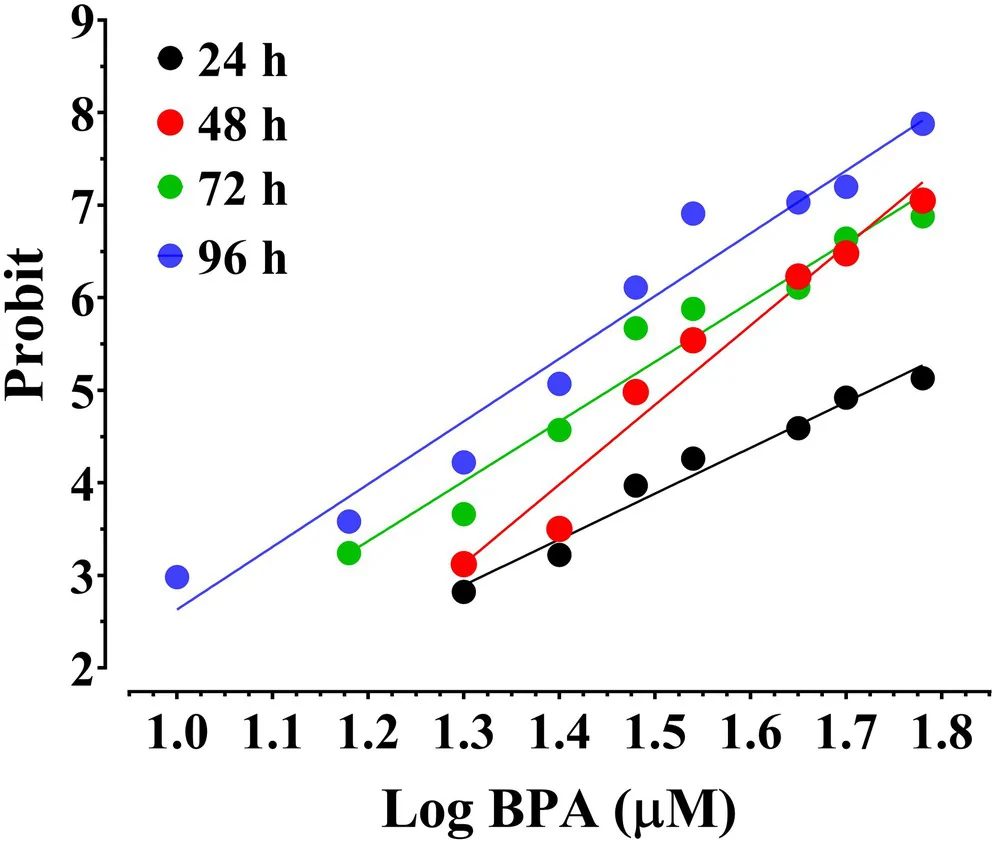
Time‐dependent acute toxicity of bisphenol A (BPA) in *Girardia tigrina* . Probit analysis of mortality after 24–96 h of exposure. The linear dose–response relationships (log‐transformed concentration vs. probit‐transformed mortality) show progressively decreasing LC_50_ values over time. Data points represent mean mortality rates from five biologically independent experiments; each performed in triplicate per concentration. Solid lines indicate the best‐fit regression models (e.g., *R* ^2^ = 0.96 at 96 h). (e.g., *R* ^2^ = 0.96 at 96 h). Full model parameters are provided in Table 1.

The mortality of *G. tigrina* in response to BPA exposure was analyzed using a probit model. The calculated LC_50_ values at 24, 48, 72, and 96 h revealed a clear time‐dependent increase in BPA toxicity (Table 1; Figure 1). At 24 h, the LC_50_ was estimated at 53.18 μM (95% CI: 27.54–138.04 μM), which decreased progressively over time: 32.99 μM at 48 h, 28.35 μM at 72 h, and 22.38 μM at 96 h (95% CI: 14.04–43.55 μM). These findings indicate that BPA exerts a cumulative toxic effect with prolonged exposure, reinforcing the suitability of *G. tigrina* as a sensitive model for assessing the environmental risks of endocrine‐disrupting compounds, such as BPA.

**TABLE 1:** Median lethal concentrations (LC_50_) of bisphenol A (BPA) in *Girardia tigrina* after 24–96 h of exposure.

Time (h)	Equation	LC_50_ (μM)	95% Confidence intervals (μM)	*R* ^2^
24	*y* = 4.951*x* − 3.544	53.18	27.54–138.04	0.9722
48	*y* = 8.595*x* − 8.050	32.99	17.21–92.71	0.9621
72	*y* = 6.460*x* − 4.383	28.35	15.75–70.08	0.9565
96	*y* = 6.780*x* − 4.152	22.38	14.04–43.55	0.9625

*Note:* Data were calculated by Probit analysis [31] with 95% confidence intervals. *R* ^2^ values indicate model fit quality. Equations represent best‐fit linear models for log‐concentration versus response. LC_50_ and 95% CI were derived via Probit analysis.

The decrease in the LC_50_ values over time illustrates the increasing lethality of the compound with extended exposure. Interestingly, the steepest slope in the probit curve was observed at 48 h (8.595), suggesting an enhanced sensitivity to BPA at this time point. Although the slope declined slightly at 72 and 96 h, the overall toxicity remained significant (Figure 1). The model showed an excellent fit to the experimental data, with *R* ^2^ values ranging from 0.9565 to 0.9722. Moreover, narrow confidence intervals reflect the precision and reliability of the estimates (Table 1). Taken together, these results underscore the time‐dependent nature of BPA toxicity and underscore the need to consider the exposure duration in ecotoxicological assessments.

**FIGURE 2:**
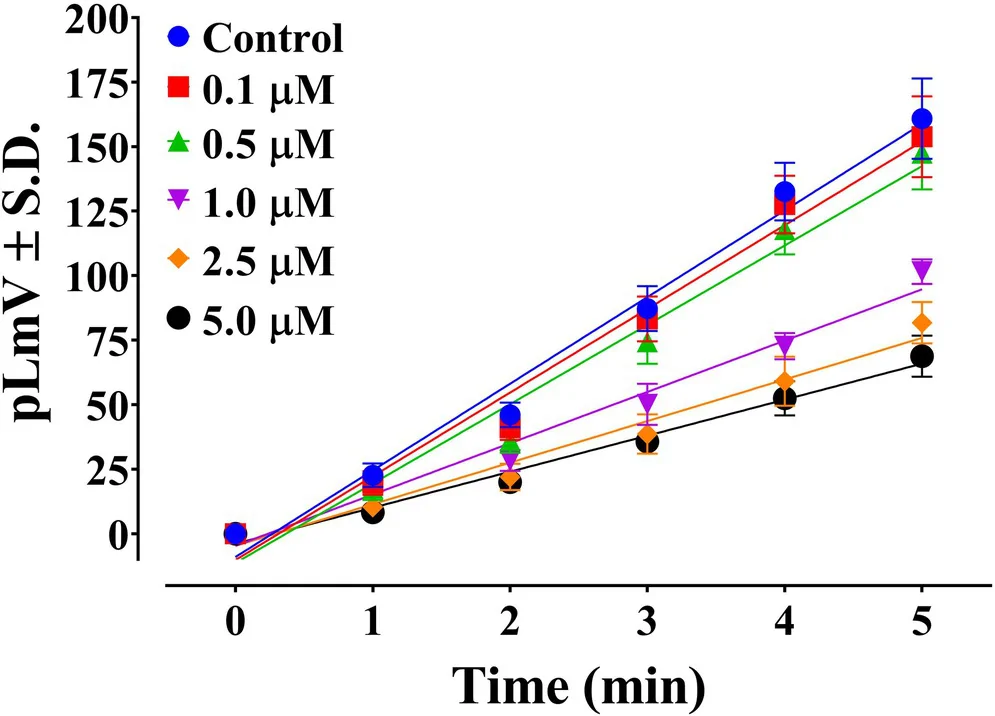
Dose‐dependent inhibition of locomotor activity in *Girardia tigrina* after 24 h of BPA exposure. Locomotion was quantified as the number of lines crossed and re‐crossed during a 5‐min observation period in polystyrene plates (BPA‐free; 60 mm diameter × 15 mm height) containing 10 mL of artificial pond water (APW). Data represent the mean ± standard deviation (SD) from five biologically independent experiments, each performed in triplicate per concentration. Statistical differences were assessed by ANOVA followed by post hoc tests (*p* < 0.05); significance markers are omitted for visual clarity.

Significant alterations in locomotor behavior were observed in *G. tigrina* after 24 h of BPA exposure (Figure 2; Table 2). At the lowest concentrations tested (0.1 and 0.5 μM), locomotion did not differ significantly from controls (adjusted *p* > 0.05), suggesting that minimal BPA exposure may not impair the neural or muscular systems responsible for basic motility. However, from 1.0 μM onward, a significant reduction in locomotor activity was detected across all time points (*p* < 0.01). The greatest impairment occurred at 5.0 μM, with a 57.2% decrease in movement at 5 min. At 1.0 μM, reductions were 23.9%, 38.5%, 42.5%, 45.2%, and 36.7% at 1, 2, 3, 4, and 5 min, respectively.

**TABLE 2:** Median inhibitory concentrations (IC_50_) of BPA for planarian locomotor velocity (pLmV) after 24 h of exposure, measured over a 5‐min test.

Time (min)	LogIC_50_	IC_50_ (μM)	95% CI (μM)	*R* ^2^
1	0.3856	2.43	1.58–3.79	0.9145
2	0.3616	2.29	1.29–4.19	0.8253
3	0.3358	2.17	1.26–3.81	0.8940
4	0.3310	2.14	1.16–4.09	0.8759
5	0.4457	2.79	1.71–4.69	0.9076

*Note:* IC_50_ values were determined by nonlinear regression. 95% confidence intervals were calculated using the profile likelihood method.

Consistently, IC_50_ values for pLmV ranged narrowly from 2.14 to 2.79 μM across the 5‐min test, indicating a sustained inhibitory effect over time (Table 2). The lowest LC_50_ was observed at 4 min (2.14 μM; 95% CI: 1.16–4.09) and the highest at 5 min (2.79 μM; 95% CI: 1.71–4.69), with overlapping confidence intervals and strong model fits (*R* ^2^ = 0.83–0.91). These results demonstrate that BPA compromises planarian motility in a dose‐ and time‐dependent manner, likely via neurotoxic mechanisms such as disruption of acetylcholine signaling and oxidative stress [49, 50, 51, 52, 53]. The temporal stability of IC_50_ values reinforces the robustness of pLmV as a sensitive behavioral endpoint for detecting sublethal neurotoxicity.

**FIGURE 3:**
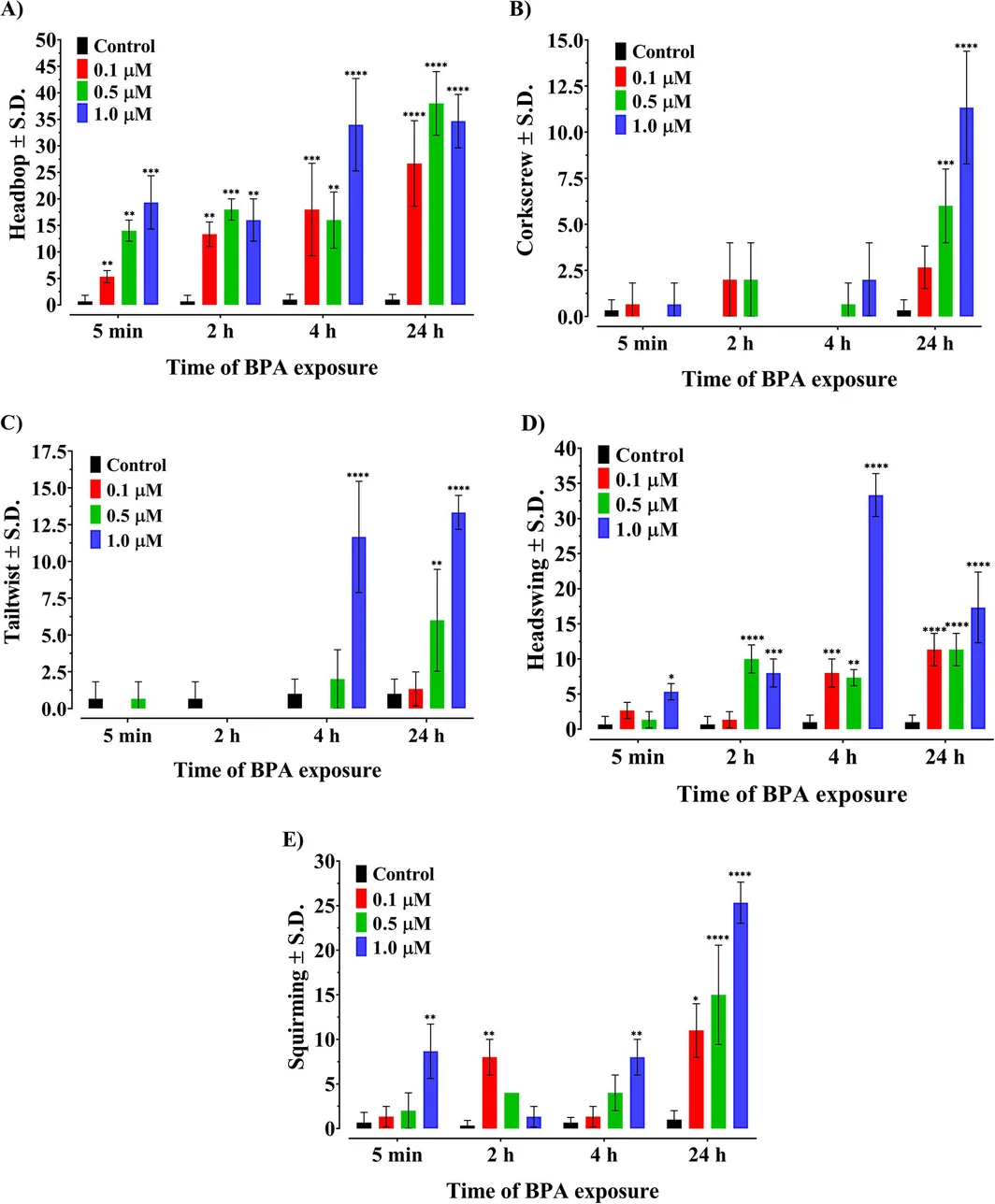
BPA‐induced seizure‐like movements (pSLMs) in *Girardia tigrina* across different exposure durations. Frequencies of (A) headbop, (B) corkscrew, (C) tail twist, (D) head swing, and (E) squirming behaviors were quantified after 5‐min, 2‐h, 4‐h, and 24‐h exposures to BPA (0.1–5.0 μM). Data represent the mean ± standard deviation (SD) from six biologically independent experiments, each performed in triplicate, with three to five planarians per treatment group. *p < 0.05, **p < 0.01, ***p < 0.001, and ****p < 0.0001 versus time‐matched controls (two‐way ANOVA followed by Dunnett's test).

In addition to locomotor deficits, BPA exposure induces stereotypical behaviors, further supporting its neurotoxic profile. Abnormal movements such as *headbop*, *corkscrew*, *tailtwist*, *headswing*, and *squirming* (Figure 3) were evaluated over time (5 min, 2 h, 4 h, and 24 h) and across BPA concentrations (0.1, 0.5, and 1.0 μM). These behaviors, absent under normal conditions, are indicative of altered neural functions [54, 55, 56].

Headbop frequency increased in a time‐ and dose‐dependent manner (Figure 3A). While 0.1 μM showed no effect at 5 min (*p* = 0.9087), both 0.5 and 1.0 μM significantly increased this behavior (*p* = 0.0196 and *p* = 0.0004, respectively). By 2 h, all concentrations produced significant effects, with marked increases persisting through 4 and 24 h. Corkscrew movements (Figure 3B) were not evident at early time points but became prominent at 24 h in response to 0.5 and 1.0 μM BPA (*p* < 0.0001). Tailtwist behavior (Figure 3C) increased significantly only at 1.0 μM after 4 h and showed dose‐dependent escalation by 24 h. Headswing (Figure 3D) was significantly induced even at 5 min with 1.0 μM (*p* = 0.0030), with effects intensifying over time. Squirming behavior (Figure 3E) followed a similar pattern: at 5 min, only 1.0 μM triggered a response (*p* = 0.0016), but by 24 h, all tested concentrations elicited significant increases (*p* < 0.0001). These stereotypies indicate the potential to disrupt neural circuits, with effects that are clearly time‐ and dose‐dependent.

**FIGURE 4:**
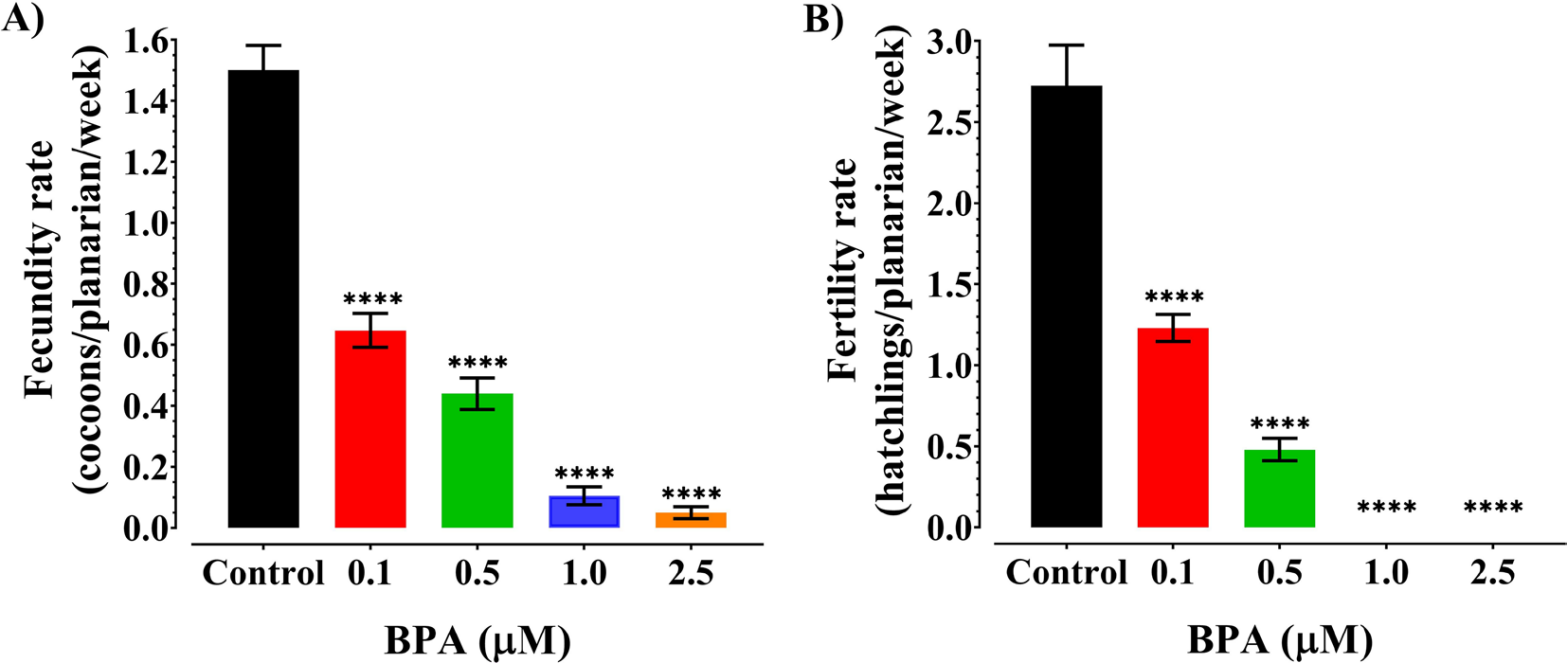
Reproductive toxicity of bisphenol A (BPA) in *Girardia tigrina* after chronic (30‐day) exposure. (A) Fecundity (cocoons per planarian per week) and (B) Fertility (hatchlings per planarian per week) were significantly reduced at concentrations ≥ 0.1 μM. Data represent the mean ± standard deviation (SD) from three biologically independent experiments, each performed in triplicate (*n* = 8 planarians per treatment). ****p < 0.0001 versus control (two‐way ANOVA followed by Dunnett's test). *Note:* Fertility data were not obtained at 1.0 and 2.5 μM due to the absence of viable hatchlings.

**FIGURE 5:**
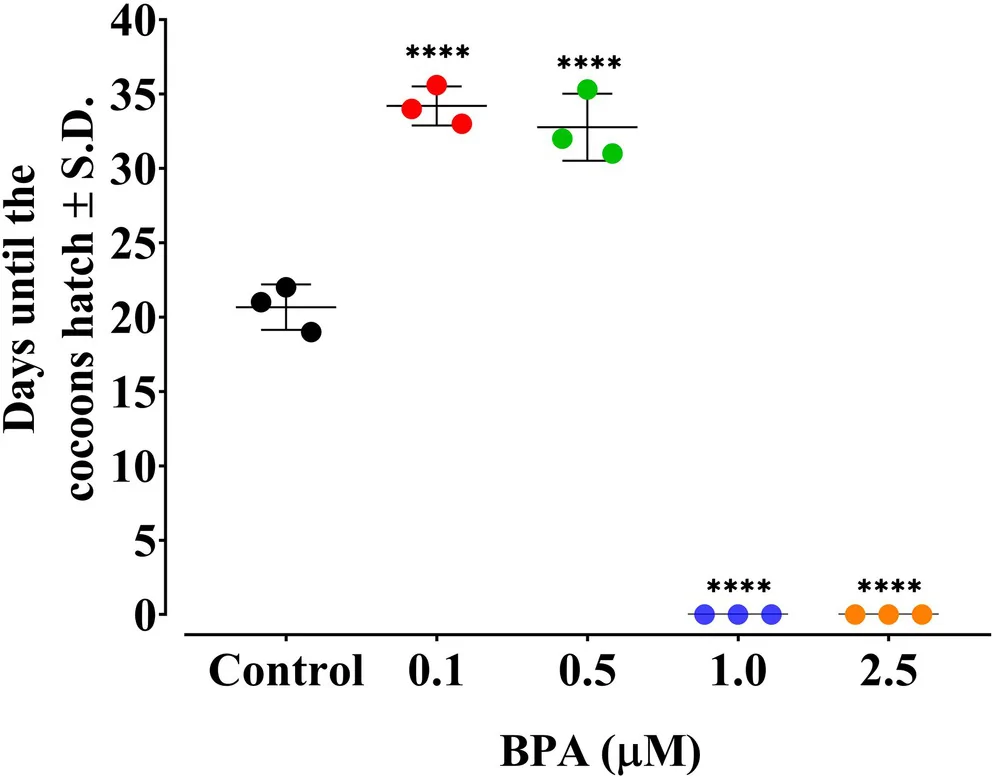
Delayed cocoon hatching in *Girardia tigrina* following chronic bisphenol A (BPA) exposure. Time to hatch (days) was significantly increased at sublethal BPA concentrations (0.1–0.5 μM) and completely inhibited at concentrations ≥ 1.0 μM (no hatchlings observed). Data represent the mean ± standard deviation (SD) from three biologically independent experiments, each performed in triplicate. ****p < 0.0001 versus control (one‐way ANOVA followed by Dunnett's test). Data points for 1.0 and 2.5 μM are shown as 0, indicating the absence of hatching.

Reproductive endpoints were also significantly affected by exposure. Two‐way ANOVA revealed a concentration‐dependent reduction in the fecundity and fertility of *G. tigrina* after BPA exposure (Figure 4A,B). The inhibitory effects were particularly striking: fecundity was reduced by 56.8%, 70.1%, 93%, and 96.7% at 0.1, 0.5, 1.0, and 2.5 μM BPA, respectively (*F*_(4,12)_ = 428.8, *p* < 0.0001). Fertility followed a similar pattern, with reductions of 55% and 82.4% at 0.1 and 0.5 μM, respectively (*F*_(4,12)_ = 328.4, *p* < 0.0001). At 1.0 and 2.5 μM, no viable hatchlings were observed 40 days post‐cocooning (Figure 5), indicating a total reproductive failure at these concentrations.

Moreover, BPA exposure significantly delayed hatching. At 0.1 and 0.5 μM, hatching time increased by an average of 13.5 days (69.5%) and 12.1 days (58.5%), respectively (*p* < 0.0001). No hatching occurred at 1.0 and 2.5 μM, reflecting 100% of the inhibition (Figure 5). These findings suggest that even low‐level exposure can impair development, whereas higher concentrations completely arrest it.

**FIGURE 6:**
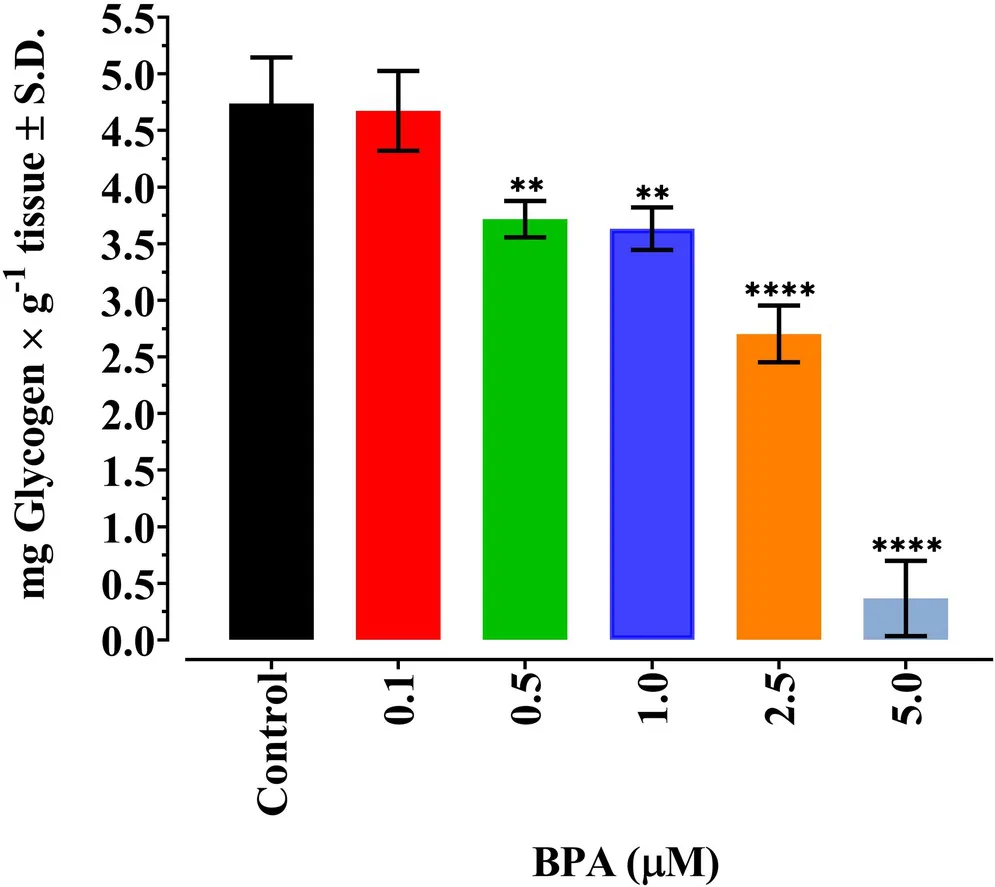
Bisphenol A‐induced glycogen depletion in *Girardia tigrina* after 7‐day exposure. Significant glycogen depletion, expressed as glucose equivalents, was observed at BPA concentrations ≥ 0.5 μM, demonstrating a dose‐dependent effect. Data represent the mean ± standard deviation (SD) from five biologically independent experiments, each performed in triplicate (*n* = 10 planarians per concentration). **p ≤ 0.0049 and ****p < 0.0001 versus control (one‐way ANOVA followed by Dunnett's post hoc test).

Disruptions in hatching dynamics appear to be closely linked to metabolic alterations, particularly in glycogen metabolism. After 7 days of BPA exposure, significant reductions in glycogen content were observed (Figure 6), beginning at 0.5 μM (*p* = 0.0049) and reaching a 21.5%, 23.3%, 42.9%, and 92.3% decrease at 0.5, 1.0, 2.5, and 5.0 μM (*p* < 0.0001), respectively. These reductions likely reflect energy deficits due to oxidative stress or endocrine disruption, potentially impairing the developmental and reproductive processes.

**FIGURE 7:**
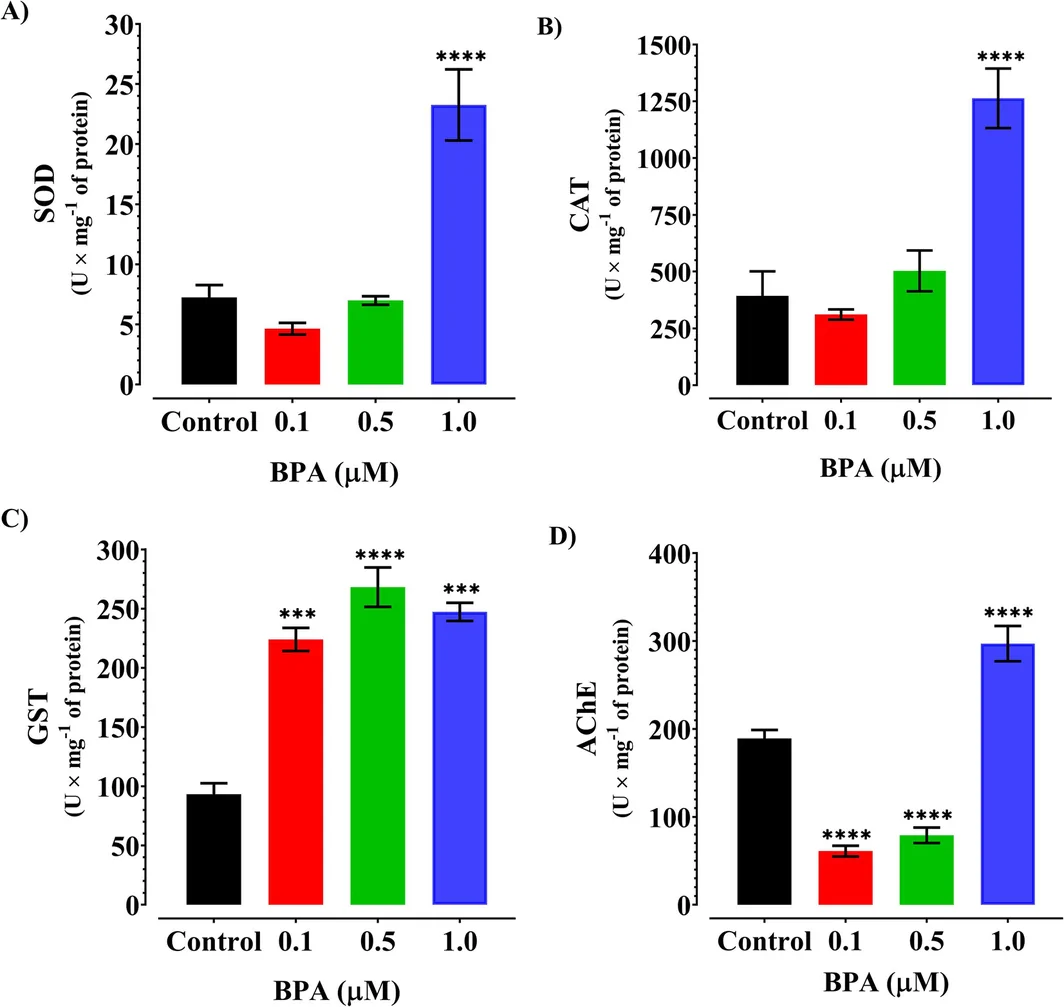
BPA modulation of antioxidant and neural enzyme activities in *Girardia tigrina* after 24 h exposure. Activities of (A) superoxide dismutase (SOD), (B) catalase (CAT), (C) glutathione‐S‐transferase (GST), and (D) acetylcholinesterase (AChE) exhibit concentration‐dependent responses. SOD and CAT activities increased significantly at 1.0 μM (****p < 0.0001), while AChE showed biphasic regulation, with inhibition at 0.1 μM and stimulation at 1.0 μM (****p < 0.0001). GST activity was significantly increased across all concentrations (***p = 0.0001 at 0.1 and 1.0 μM; ****p < 0.0001 at 0.5 μM). Data represent the mean ± standard deviation (SD) from three biologically independent experiments performed in triplicate. Statistical analysis was performed using one‐way ANOVA followed by Dunnett's post hoc test.

To further investigate metabolic stress, we analyzed the activities of key enzymes: SOD, CAT, GST, and AChE (Figure 7). A significant effect of BPA exposure on SOD activity was observed (*F*_(3,16)_ = 128.3, *p* < 0.0001, *R* ^2^ = 0.96). While no statistically significant differences were found at 0.1 and 0.5 μM (*p* = 0.0946), a trend toward increased SOD activity was noted at the lowest concentration. A marked induction statistically significant occurred at 1.0 μM (*p* < 0.0001), supporting the role of BPA‐induced oxidative stress in mediating the observed physiological disturbances.

At 1.0 μM BPA, CAT activity was significantly inhibited (*p* < 0.0001), suggesting compromised H_2_O_2_ detoxification capacity. Lower concentrations (0.1 and 0.5 μM) had no discernible impact on the enzyme function. Statistical analysis supported a robust treatment effect (*F*_(3,8)_ = 50.99, *p* < 0.0001, *R* ^2^ = 0.95), with homogeneous variances (Brown–Forsythe *p* = 0.5327).

GST activity was significantly stimulated across all BPA concentrations tested (0.1–1.0 μM), with the highest response observed at 0.5 μM (*p* < 0.0001). This response profile indicates a dose‐responsive enhancement of the detoxification capacity, likely reflecting adaptive activation of phase II metabolism. ANOVA revealed a pronounced treatment effect (*F*_(3,5)_ = 101.6, *p* < 0.0001, *R* ^2^ = 0.984), with homogeneous variances among groups (Brown–Forsythe *p* = 0.4227).

Non‐monotonic modulation of AChE was observed. At 0.1 (−67.8%) and 0.5 μM (−58.2%), BPA significantly inhibited enzyme activity (*p* < 0.0001), whereas a marked increase was observed at 1.0 μM (+56.9%) (*p* < 0.0001). This biphasic response pattern is typical of endocrine disruptors. Statistical analysis confirmed the robustness of the treatment effect (*F*_(3,10)_ = 275.7, *p* < 0.0001, *R* ^2^ = 0.988), with consistent variances (Brown–Forsythe *p* = 0.8171).

The enzymatic responses of *G. tigrina* after 24‐h exposure to BPA demonstrated the coordinated activation of antioxidant and neurofunctional pathways, suggesting a systemic biochemical response to xenobiotic stress. SOD activity sharply increased at 1.0 μM BPA, indicating elevated superoxide radical generation and activation of the primary antioxidant defense. Similarly, CAT activity was also significantly upregulated at this concentration, reflecting enhanced breakdown of hydrogen peroxide as part of a compensatory antioxidant response. GST activity was markedly stimulated at all tested concentrations, including the lowest (0.1 μM), reflecting early and persistent activation of phase II detoxification pathways in response to oxidative by‐products or electrophilic BPA metabolites. AChE showed a non‐monotonic response: inhibited at 0.1 and 0.5 μM, and significantly induced at 1.0 μM, consistent with a dual‐phase neurotoxic effect commonly associated with EDCs. The concurrent activation of SOD, CAT, GST, and AChE at the highest BPA concentration suggests a coordinated redox response accompanied by altered cholinergic signaling. These findings reveal that even submicromolar levels of BPA can trigger complex oxidative and neurochemical responses in planarians, reinforcing their utility as sensitive bioindicators of environmental toxicity.

## 4 Discussion

The present study demonstrates that *G. tigrina* is highly sensitive to BPA exposure, with both lethal and sublethal endpoints revealing a clear time‐ and dose‐dependent pattern of toxicity. The progressive decrease in LC_50_ values over 96 h suggests cumulative effects of BPA and reinforces the importance of considering exposure duration in environmental risk assessments. Similar time‐dependent increases in toxicity have been reported in other aquatic invertebrates, emphasizing that chronic, low‐level exposures may be as ecologically harmful as acute events.

The relatively high *R* ^2^ values obtained across all time points on the pLmV experiment further support the consistency and quality of the nonlinear regression model. These findings align with previous reports indicating that sublethal neurotoxicants such as BPA can impair serotonergic and dopaminergic signaling pathways involved in planarian motor control [57, 58]. Therefore, the early onset of locomotor inhibition may reflect the rapid bioavailability of BPA and its prompt interference with neurotransmission. In ecotoxicological terms, the narrow IC_50_ interval and its stability suggest that even brief behavioral assays may be sufficient to detect BPA‐induced neuroactivity, potentially reducing the need for extended exposure times or longer behavioral tracking in future bioassays.

Behavioral endpoints, particularly locomotor inhibition and the emergence of stereotypical movements, indicate that BPA likely disrupts neuronal signaling pathways in planarians. These findings are consistent with previous studies that have identified AChE inhibition and oxidative stress as key mechanisms underlying BPA‐induced neurotoxicity [21]. The early onset of behaviors such as headswing and squirming further supports the hypothesis that neural circuits are rapidly and persistently affected, even at micromolar concentrations. Given that planarian behavior is tightly coupled to central nervous system function, such changes may serve as reliable indicators of neurotoxic stress in freshwater ecosystems.

Recent studies have increasingly validated freshwater planarians as valuable in vivo bioindicators for ecotoxicological assessments due to their ecological relevance, neurobehavioral complexity, and regenerative capabilities. Their capacity to integrate multiple biological responses, ranging from neurobehavioral to metabolic and reproductive endpoints, makes them particularly suited for rapid and cost‐effective environmental screening [21, 22]. For instance, standardized behavioral tests using planarians have demonstrated high sensitivity to a range of contaminants, such as phenolic compounds and heavy metals, and can be completed within hours, facilitating high‐throughput screening efforts [21, 59]. Furthermore, their ability to regenerate a functional brain offers unique opportunities for evaluating neurotoxicity and recovery following pollutant exposure [60], while recent approaches have applied planarian regeneration to detect sublethal pollution levels in water samples [61].

While planarians offer significant advantages as alternative models for rapid and integrative toxicity assessment, extrapolating their responses to vertebrate systems requires careful consideration. Differences in physiology, xenobiotic metabolism, hormonal regulation, and neural architecture may influence how organisms respond to environmental contaminants like BPA. For instance, while both planarians and vertebrates possess cholinergic and oxidative stress pathways, the complexity of vertebrate endocrine and reproductive systems introduces additional layers of regulation that may alter susceptibility or adaptive responses. Therefore, complementary studies using established vertebrate models, such as fish embryos or amphibians, are necessary to validate the ecological and regulatory relevance of planarian‐based findings. Such cross‐taxa comparisons can bridge mechanistic insights and support more robust environmental risk assessments.

The impairment of reproductive capacity observed in this study is particularly concerning. Significant reductions in fecundity and fertility, accompanied by delayed or failed hatching, suggest that BPA can disrupt hormonal and developmental pathways critical for species survival. These effects parallel those described for other EDCs, reinforcing BPA's role as a potent reproductive toxicant. Moreover, the observed depletion of glycogen reserves and the dysregulation of key enzymes, such as SOD, CAT, and AChE, indicate that oxidative stress and metabolic dysfunction are integral to BPA's toxic action. These physiological disruptions likely contribute to the observed deficits in reproduction, development, and behavior.

To deepen the mechanistic understanding of BPA‐induced toxicity, future research should incorporate transcriptomic approaches, such as RNA sequencing or targeted gene expression profiling. These tools can provide a systems‐level view of molecular pathways disrupted by exposure, including those involved in oxidative stress, neurotransmission, endocrine signaling, and regeneration. Integrating transcriptomic data with behavioral, biochemical, and reproductive endpoints can help elucidate the upstream regulatory networks and downstream phenotypic outcomes, ultimately enhancing the predictive value of planarian‐based assays. Moreover, such molecular analyses could facilitate the identification of novel biomarkers and toxicity signatures relevant for environmental monitoring and regulatory decision‐making.

Altogether, the results highlight *G. tigrina* as a robust model for integrated ecotoxicological assessments, capable of detecting neurobehavioral, metabolic, and reproductive alterations. Given BPA's widespread presence in aquatic environments and the increasing regulatory restrictions on its use in Europe and other regions, our findings provide timely evidence in support of stricter environmental monitoring and risk management strategies, especially in freshwater systems where invertebrate species play key ecological roles in maintaining ecosystem stability.

Despite the strength of the multilevel assessment conducted in this study, several limitations should be acknowledged. First, the use of a single model organism, *G. tigrina* , while advantageous for regenerative and behavioral endpoints, limits the extrapolation of findings to other taxa, including vertebrates and species with distinct metabolic or endocrine profiles. Second, the experimental exposures were conducted under controlled laboratory conditions, which may not fully replicate the complexity of natural aquatic environments, where interactions with other contaminants or fluctuating abiotic factors (e.g., temperature, pH, and light) may modulate BPA toxicity. Additionally, molecular endpoints such as gene expression or receptor‐binding assays were not included; thus, the mechanistic understanding of BPA's action remains primarily inferential, based on enzymatic and behavioral responses. Another important limitation is the relatively short exposure duration employed in the present study. While the 24‐ to 96‐h exposures effectively revealed acute and sublethal effects of BPA on multiple physiological systems, they may not fully capture persistent or delayed responses that arise from chronic or developmental exposures. Real‐world aquatic environments often involve continuous or intermittent contaminant inputs over extended periods, which can lead to bioaccumulation, adaptive responses, or multigenerational effects. Therefore, future studies should prioritize long‐term and transgenerational assessments of BPA toxicity under semi‐natural or flow‐through conditions to better simulate ecological realism and evaluate lasting impacts on regeneration, reproduction, and population dynamics. Future studies incorporating transcriptomic or proteomic analyses, as well as field validation, are warranted to complement and extend the present findings.

Despite regulatory efforts, BPA remains a contaminant of concern due to its widespread environmental persistence and continued human exposure. Recent biomonitoring studies have reported detectable levels of BPA in urine, serum, and even placental tissues across diverse populations, including in regions with partial regulatory bans [62, 63]. Moreover, low‐dose chronic exposure has been linked to subtle but significant disruptions in neurodevelopment, reproductive function, and metabolic regulation, particularly during early life stages [8, 64]. These findings suggest that current regulatory thresholds may not fully protect against long‐term health risks, especially in vulnerable groups. Therefore, further investigation into BPA's effects on key physiological endpoints using alternative, sensitive models remains both scientifically and socially relevant. The use of freshwater planarians, which integrate neurobehavioral, reproductive, and metabolic responses, offers a valuable tool for evaluating the sublethal impacts of BPA at environmentally realistic concentrations.

## 5 Conclusion

This study clearly demonstrates that BPA induces marked neurotoxic, metabolic, and reproductive disturbances in *G. tigrina* at environmentally relevant concentrations ranging from 0.1 to 5.0 μM. The dose‐ and time‐dependent responses observed across behavioral metrics (including locomotor deficits and seizure‐like activity), physiological indicators (particularly glycogen depletion), and reproductive outcomes (such as decreased cocoon viability) underscore the compound's capacity to disrupt multiple, interconnected biological systems in freshwater invertebrates.

Importantly, the pronounced sensitivity of planarians to sublethal exposures, especially below 1.0 μM, highlights a significant limitation of traditional ecotoxicological assays focused solely on lethality. Such tests may fail to capture subtle yet ecologically significant effects. These findings reinforce the value of integrating behavioral, biochemical, and reproductive biomarkers into contemporary environmental risk assessment protocols.

Moreover, the structural similarity between BPA and widely used industrial alternatives such as BPS and BPF raises critical concerns regarding the potential for shared toxicological pathways. In the absence of rigorous pre‐market toxicological evaluation, these substitutes may pose environmental risks comparable to those associated with BPA. Our results therefore support the implementation of precautionary regulatory frameworks that mandate sublethal toxicity screening for emerging contaminants, alongside efforts to improve water treatment technologies aimed at reducing pollutant loads in aquatic environments.

Future studies should prioritize the investigation of chronic and transgenerational effects of low‐dose exposures, particularly in model organisms such as planarians, which offer valuable insights into regeneration, reproduction, and developmental plasticity. Such research will be essential for deepening our understanding of the long‐term impacts of EDCs and for informing effective mitigation strategies to safeguard ecosystem integrity and public health.

## Author Contributions

R.d.J.S. and F.S. performed the experiments, obtained the full data for the study, and took responsibility for the integrity of the data and the accuracy of the data analyses. W.L.B. conceived and designed the study. R.d.F.S., F.S., and W.L.B. wrote the manuscript. R.d.F.S., F.S., and W.L.B. revised the manuscript critically and performed statistical analyses. All authors approved the final version of this manuscript.

## Funding

The authors have nothing to report.

## Conflicts of Interest

The authors declare no conflicts of interest.

## Data Availability

The data that support the findings of this study are available from the corresponding author upon request.

## References

[1] T. Geens , L. Goeyens , and A. Covaci , “Are Potential Sources for Human Exposure to Bisphenol‐A Overlooked?,” International Journal of Hygiene and Environmental Health 214, no. 5 (2011): 339–347, 10.1016/J.IJHEH.2011.04.005. 21570349

[2] Y. Q. Huang , C. K. Wong , J. S. Zheng , et al., “Bisphenol A (BPA) in China: A Review of Sources, Environmental Levels, and Potential Human Health Impacts,” Environment International 42 (2012): 91–99, 10.1016/j.envint.2011.04.010. 21596439

[3] E. S. Ihde , S. Zamudio , J. M. Loh , et al., “Application of a Novel Mass Spectrometric (MS) Method to Examine Exposure to Bisphenol‐A and Common Substitutes in a Maternal Fetal Cohort,” Human and Ecological Risk Assessment 24, no. 2 (2017): 331–346, 10.1080/10807039.2017.1381831. 31588171 PMC6777866

[4] A. V. Krishnan , P. Stathis , S. F. Permuth , L. Tokes , and D. Feldman , “Bisphenol‐A: An Estrogenic Substance Is Released From Polycarbonate Flasks During Autoclaving,” Endocrinology 132, no. 6 (1993): 2279–2286, 10.1210/ENDO.132.6.8504731. 8504731

[5] J. H. Kang , F. Kondo , and Y. Katayama , “Human Exposure to Bisphenol A,” Toxicology 226, no. 2–3 (2006): 79–89, 10.1016/J.TOX.2006.06.009. 16860916

[6] C. Kubwabo , I. Kosarac , B. Stewart , B. R. Gauthier , K. Lalonde , and P. J. Lalonde , “Migration of Bisphenol A From Plastic Baby Bottles, Baby Bottle Liners and Reusable Polycarbonate Drinking Bottles,” Food Additives & Contaminants. Part A, Chemistry, Analysis, Control, Exposure & Risk Assessment 26, no. 6 (2009): 928–937, 10.1080/02652030802706725. 19680968

[7] V. Robles‐Aguilera , Y. Gálvez‐Ontiveros , L. Rodrigo , et al., “Factors Associated With Exposure to Dietary Bisphenols in Adolescents,” Nutrients 13, no. 5 (2021): 1–13, 10.3390/NU13051553. PMC8147950 34062990

[8] C. Lambré , J. M. Barat Baviera , C. Bolognesi , et al., “Re‐Evaluation of the Risks to Public Health Related to the Presence of Bisphenol A (BPA) in Foodstuffs,” EFSA Journal 21, no. 4 (2023): e06857, 10.2903/J.EFSA.2023.6857. 37089179 PMC10113887

[9] S. B. Gewurtz , G. Tardif , M. Power , et al., “Bisphenol A in the Canadian Environment: A Multimedia Analysis,” Science of the Total Environment 755 (2021): 142472, 10.1016/J.SCITOTENV.2020.142472. 33059142

[10] J. Wang , F. K. S. Chan , M. F. Johnson , et al., “Material Cycles, Environmental Emissions, and Ecological Risks of Bisphenol A (BPA) in China and Implications for Sustainable Plastic Management,” Environmental Science & Technology 59, no. 3 (2025): 1631–1646, 10.1021/ACS.EST.4C09876/SUPPL_FILE/ES4C09876_SI_001.PDF. 39723815 PMC11780737

[11] J. Liang , C. Li , Y. Dang , et al., “Occurrence of Bisphenol A Analogues in the Aquatic Environment and Their Behaviors and Toxicity Effects in Plants,” Environment International 193 (2024): 109105, 10.1016/J.ENVINT.2024.109105. 39489000

[12] The European Parliament and the Council of the European Union , “Directive—2020/2184—EN—EUR‐Lex,” Directive (EU) 2020/2184 of the European Parliament and of the Council of 16 December 2020 on the Quality of Water Intended for Human Consumption, 2020, accessed July 8, 2025, https://eur‐lex.europa.eu/legal‐content/EN/TXT/?uri=CELEX:32020L2184.

[13] J. Corrales , L. A. Kristofco , W. B. Steele , et al., “Global Assessment of Bisphenol A in the Environment: Review and Analysis of Its Occurrence and Bioaccumulation,” Dose‐Response 13, no. 3 (2015): 1559325815598308, 10.1177/1559325815598308. 26674671 PMC4674187

[14] P. Loganathan , S. Vigneswaran , J. Kandasamy , T. V. Nguyen , A. Katarzyna Cuprys , and H. Ratnaweera , “Bisphenols in Water: Occurrence, Effects, and Mitigation Strategies,” Chemosphere 328 (2023): 138560, 10.1016/J.CHEMOSPHERE.2023.138560. 37004822

[15] J. d. S. Santos , M. d. S. Pontes , M. B. de Souza , et al., “Toxicity of Bisphenol A (BPA) and Its Analogues BPF and BPS on the Free‐Floating Macrophyte *Salvinia biloba* ,” Chemosphere 343 (2023): 1–12, 10.1016/J.CHEMOSPHERE.2023.140235. 37734497

[16] J. R. Rochester , A. L. Bolden , and C. F. Kwiatkowski , “Prenatal Exposure to Bisphenol A and Hyperactivity in Children: A Systematic Review and Meta‐Analysis,” Environment International 114 (2018): 343–356, 10.1016/J.ENVINT.2017.12.028. 29525285

[17] H. Wang , R. Gao , W. Liang , et al., “Large‐Scale Biomonitoring of Bisphenol Analogues and Their Metabolites in Human Urine From Guangzhou, China: Implications for Health Risk Assessment,” Chemosphere 338 (2023): 139601, 10.1016/J.CHEMOSPHERE.2023.139601. 37480947

[18] J. Liu , L. Zhang , G. Lu , R. Jiang , Z. Yan , and Y. Li , “Occurrence, Toxicity and Ecological Risk of Bisphenol A Analogues in Aquatic Environment—A Review,” Ecotoxicology and Environmental Safety 208 (2021): 111481, 10.1016/J.ECOENV.2020.111481. 33120264

[19] J. B. Best and M. Morita , “Toxicology of Planarians,” Hydrobiologia 227, no. 1 (1991): 375–383, 10.1007/BF00027626.

[20] J. P. Wu and M. H. Li , “The Use of Freshwater Planarians in Environmental Toxicology Studies: Advantages and Potential,” Ecotoxicology and Environmental Safety 161 (2018): 45–56, 10.1016/J.ECOENV.2018.05.057. 29859407

[21] D. Hagstrom , O. Cochet‐Escartin , S. Zhang , C. Khuu , and E. M. S. Collins , “Freshwater Planarians as an Alternative Animal Model for Neurotoxicology,” Toxicological Sciences 147, no. 1 (2015): 270, 10.1093/TOXSCI/KFV129. 26116028 PMC4838007

[22] T. Knakievicz and H. B. Ferreira , “Evaluation of Copper Effects Upon *Girardia tigrina* Freshwater Planarians Based on a Set of Biomarkers,” Chemosphere 71, no. 3 (2008): 419–428, 10.1016/j.chemosphere.2007.11.004. 18078977

[23] M. E. Deochand , T. R. Birkholz , and W. S. Beane , “Temporal Regulation of Planarian Eye Regeneration,” Regeneration 3, no. 4 (2016): 209–221, 10.1002/REG2.61. 27800171 PMC5084360

[24] F. R. Buttarelli , C. Pellicano , and F. E. Pontieri , “Neuropharmacology and Behavior in Planarians: Translations to Mammals,” Comparative Biochemistry and Physiology Part C: Toxicology & Pharmacology 147, no. 4 (2008): 399–408, 10.1016/j.cbpc.2008.01.009. 18294919

[25] F. Cebrià , C. Kobayashi , Y. Umesono , et al., “FGFR‐Related Gene Nou‐Darake Restricts Brain Tissues to the Head Region of Planarians,” Nature 419, no. 6907 (2002): 620–624, 10.1038/NATURE01042. 12374980

[26] F. Schaly and W. L. Braguini , “Assessment of Aluminium Toxicity on *Dugesia tigrina*: Implications for Reproduction, Behaviour, and Enzymatic Metabolism,” Chemical Ecology 40 (2024): 515–534, 10.1080/02757540.2024.2335330.

[27] P. U. Ofoegbu , F. C. P. Simão , A. Cruz , S. Mendo , A. M. V. M. Soares , and J. L. T. Pestana , “Toxicity of Tributyltin (TBT) to the Freshwater Planarian *Schmidtea mediterranea* ,” Chemosphere 148 (2016): 61–67, 10.1016/J.CHEMOSPHERE.2015.12.131. 26802264

[28] A. Kim and S. M. Rawls , “Nicotine‐Induced C‐Shape Movements in Planarians Are Reduced by Antinociceptive Drugs: Implications for Pain in Planarian Paroxysm Etiology?,” Brain Research 1778 (2022): 147770, 10.1016/J.BRAINRES.2021.147770. 34979130 PMC8816893

[29] E. Ted and J. Mcconnell , A Manual of Psychological Experimentation on Planarians (Mental Health Research Institute, The University of Michigan, 1965).

[30] ASTM , ASTM International ‐ Standards Worldwide (American Society for Testing and Materials, 1980), 406–430.

[31] D. J. Finney , “Probit Analysis,” Journal of Pharmaceutical Sciences 60, no. 9 (1971): 1432, 10.1002/jps.2600600940.

[32] G. M. Klečka , C. A. Staples , K. E. Clark , N. Van Der Hoeven , D. E. Thomas , and S. G. Hentges , “Exposure Analysis of Bisphenol A in Surface Water Systems in North America and Europe,” Environmental Science & Technology 43, no. 16 (2009): 6145–6150, 10.1021/ES900598E. 19746705

[33] J. B. Best and M. Morita , “Toxicology of Planarians,” in Turbellarian Biology, vol. 227 (Kluwer Academic Publishers Group, 1991), 375–383, 10.1007/978-94-011-2775-2_53.

[34] R. B. Raffa and P. Desai , “Description and Quantification of Cocaine Withdrawal Signs in Planaria,” Brain Research 1032, no. 1–2 (2005): 200–202, 10.1016/J.BRAINRES.2004.10.052. 15680960

[35] O. R. Pagán , D. Baker , S. Deats , et al., “Planarians in Pharmacology: Parthenolide Is a Specific Behavioral Antagonist of Cocaine in the Planarian *Girardia tigrina* ,” International Journal of Developmental Biology 56, no. 1–3 (2012): 193–196, 10.1387/IJDB.113486OP. 22451007

[36] R. Bakeman and J. M. Gottman , Observing Interaction (Cambridge University Press, 1997), 10.1017/CBO9780511527685.

[37] P. Martin and P. Bateson , Measuring Behaviour: An Introductory Guide (Cambridge University Press, 2007), 10.1017/CBO9780511810893.

[38] M. W. Watkins and M. Pacheco , “Interobserver Agreement in Behavioral Research: Importance and Calculation,” Journal of Behavioral Education 10, no. 4 (2000): 205–212, 10.1023/A:1012295615144/METRICS.

[39] J. R. Landis and G. G. Koch , “The Measurement of Observer Agreement for Categorical Data,” Biometrics 33, no. 1 (1977): 159, 10.2307/2529310. 843571

[40] T. Knakievicz , S. M. Vieira , B. Erdtmann , and H. B. Ferreira , “Reproductive Modes and Life Cycles of Freshwater Planarians (Platyhelminthes, Tricladida, Paludicula) From Southern Brazil,” Invertebrate Biology 125 (2006): 212–221, 10.1111/j.1744-7410.2006.00054.x.

[41] A. Kemp and A. J. Van Heijningen , “A Colorimetric Micro‐Method for the Determination of Glycogen in Tissues,” Biochemical Journal 56 (1954): 646–648, 10.1042/bj0560646. 13159896 PMC1269684

[42] Y. Kono , “Generation of Superoxide Radical During Autoxidation of Hydroxylamine and an Assay for Superoxide Dismutase,” Archives of Biochemistry and Biophysics 186, no. 1 (1978): 189–195, 10.1016/0003-9861(78)90479-4. 24422

[43] E. F. Elstner and A. Heupel , “Inhibition of Nitrite Formation From Hydroxylammoniumchloride: A Simple Assay for Superoxide Dismutase,” Analytical Biochemistry 70, no. 2 (1976): 616–620, 10.1016/0003-2697(76)90488-7. 817618

[44] H. Aebi , “Catalase In Vitro,” Methods in Enzymology 105 (1984): 121–126, 10.1016/S0076-6879(84)05016-3. 6727660

[45] Y. Li and H. E. Schellhorn , “Rapid Kinetic Microassay for Catalase Activity,” ABRF Journal of Biomolecular Technology 18, no. 4 (2007): 185–187. PMC2062561 17916790

[46] W. Habig , M. Pabst , and W. Jakoby , “Glutathione S‐Transferases. The First Enzymatic Step in Mercapturic Acid Formation,” Journal of Biological Chemistry 249, no. 22 (1974): 7130–7139, 10.1016/S0021-9258(19)42083-8. 4436300

[47] G. L. Ellman , K. D. Courtney , V. Andres , and R. M. Featherstone , “A New and Rapid Colorimetric Determination of Acetylcholinesterase Activity,” Biochemical Pharmacology 7, no. 2 (1961): 88–95, 10.1016/0006-2952(61)90145-9. 13726518

[48] M. M. Bradford , “A Rapid and Sensitive Method for the Quantitation of Microgram Quantities of Protein Utilizing the Principle of Protein‐Dye Binding,” Analytical Biochemistry 72 (1976): 248–254, 10.1016/0003-2697(76)90527-3. 942051

[49] J. Mathieu‐Denoncourt , S. J. Wallace , S. R. de Solla , and V. S. Langlois , “Influence of Lipophilicity on the Toxicity of Bisphenol A and Phthalates to Aquatic Organisms,” Bulletin of Environmental Contamination and Toxicology 97, no. 1 (2016): 4–10, 10.1007/S00128-016-1812-9/TABLES/2. 27169527

[50] S. Tando , K. Itoh , T. Yaoi , J. Ikeda , Y. Fujiwara , and S. Fushiki , “Effects of Pre‐ and Neonatal Exposure to Bisphenol A on Murine Brain Development,” Brain & Development 29, no. 6 (2007): 352–356, 10.1016/j.braindev.2006.10.003. 17113258

[51] S. Tao , Y. Zhang , C. Yuan , J. Gao , F. Wu , and Z. Wang , “Oxidative Stress and Immunotoxic Effects of Bisphenol A on the Larvae of Rare Minnow *Gobiocypris rarus* ,” Ecotoxicology and Environmental Safety 124 (2016): 377–385, 10.1016/J.ECOENV.2015.11.014. 26595511

[52] X. Wang , Q. Dong , Y. Chen , et al., “Bisphenol A Affects Axonal Growth, Musculature and Motor Behavior in Developing Zebrafish,” Aquatic Toxicology 142–143 (2013): 104–113, 10.1016/J.AQUATOX.2013.07.011. 23994041

[53] Y. Wang , J. Wu , D. Wang , et al., “BPA Induces Hepatotoxicity in Zebrafish Through Oxidative Stress and Apoptosis Pathways,” Fish Physiology and Biochemistry 50, no. 2 (2024): 403–412, 10.1007/S10695-023-01284-4. 38085449

[54] K. Ouyang , S. Nayak , Y. Lee , et al., “Behavioral Effects of Splenda, Equal and Sucrose: Clues From Planarians on Sweeteners,” Neuroscience Letters 636 (2017): 213–217, 10.1016/j.neulet.2016.11.017. 27845240 PMC5148653

[55] F. R. Buttarelli , F. E. Pontieri , V. Margotta , and G. Palladini , “Acetylcholine/Dopamine Interaction in Planaria,” Comparative Biochemistry and Physiology Part C: Toxicology & Pharmacology 125, no. 2 (2000): 225–231, 10.1016/S0742-8413(99)00111-5. 11790344

[56] S. M. Rawls , T. Patil , C. S. Tallarida , et al., “Nicotine Behavioral Pharmacology: Clues From Planarians,” Drug and Alcohol Dependence 118, no. 2–3 (2011): 274–279, 10.1016/j.drugalcdep.2011.04.001. 21530106 PMC3163013

[57] J. B. Best and M. Morita , “Planarians as a Model System for In Vitro Teratogenesis Studies,” Teratogenesis, Carcinogenesis, and Mutagenesis 3 (1982): 277, 10.1002/1520-6866(1990)2:3/4. 6130627

[58] K. Nishimura , Y. Kitamura , T. Taniguchi , and K. Agata , “Analysis of Motor Function Modulated by Cholinergic Neurons in Planarian *Dugesia japonica* ,” Neuroscience 168, no. 1 (2010): 18–30, 10.1016/J.NEUROSCIENCE.2010.03.038. 20338223

[59] Z. Cao , F. Wang , C. Xiu , J. Zhang , and Y. Li , “ *Hypericum perforatum* Extract Attenuates Behavioral, Biochemical, and Neurochemical Abnormalities in Aluminum Chloride‐Induced Alzheimer's Disease Rats,” Biomedicine & Pharmacotherapy 91 (2017): 931–937, 10.1016/J.BIOPHA.2017.05.022. 28514831

[60] R. R. Sousa , R. B. Vasconcelos , R. S. Barbosa , et al., “Behavioral and Physiological Responses of *Girardia tigrina* Exposed to Polyethylene Microplastics,” Environmental Science and Pollution Research 31, no. 33 (2024): 46052–46060, 10.1007/S11356-024-34304-8. 38981965

[61] T. Inoue , H. Hoshino , T. Yamashita , S. Shimoyama , and K. Agata , “Planarian Shows Decision‐Making Behavior in Response to Multiple Stimuli by Integrative Brain Function,” Zoological Letters 1 (2015): 1–15, 10.1186/s40851-014-0010-z. 26605052 PMC4657317

[62] S. Moon , S. H. Yu , C. B. Lee , Y. J. Park , H. J. Yoo , and D. S. Kim , “Effects of Bisphenol A on Cardiovascular Disease: An Epidemiological Study Using National Health and Nutrition Examination Survey 2003–2016 and Meta‐Analysis,” Science of the Total Environment 763 (2021): 142941, 10.1016/J.SCITOTENV.2020.142941. 33158523

[63] X. Ye , L. Y. Wong , J. Kramer , X. Zhou , T. Jia , and A. M. Calafat , “Urinary Concentrations of Bisphenol A and Three Other Bisphenols in Convenience Samples of U.S. Adults During 2000‐2014,” Environmental Science & Technology 49, no. 19 (2015): 11834–11839, 10.1021/ACS.EST.5B02135. 26360019 PMC7948051

[64] J. R. Rochester and A. L. Bolden , “Bisphenol S and F: A Systematic Review and Comparison of the Hormonal Activity of Bisphenol a Substitutes,” Environmental Health Perspectives 123, no. 7 (2015): 643–650, 10.1289/EHP.1408989. 25775505 PMC4492270

